# Comparison of 1L Adjuvant Auxiliary Preparations with 2L Solely Polyethylene Glycol plus Ascorbic Acid Regime for Bowel Cleaning: A Meta-analysis of Randomized, Controlled Trials

**DOI:** 10.1155/2021/6638858

**Published:** 2021-02-18

**Authors:** Xin Yuan, Zhixin Zhang, Jiarong Xie, Yu Zhang, Lu Xu, Weihong Wang, Lei Xu

**Affiliations:** ^1^College of Medicine, Ningbo University, Zhejiang, China; ^2^Department of Gastroenterology, Ningbo First Hospital, Zhejiang, China; ^3^Clinical Department for Intensive Care, Ningbo No.2 Hospital, Zhejiang, China

## Abstract

The effectiveness of additional usage of adjuvants for bowel preparation is still unclear. This study compared 1L polyethylene glycol plus ascorbic acid with adjuvant drug regimens (1L PEG-AA, lower volume) with 2L polyethylene glycol plus ascorbic acid (2L PEG-A, low volume) to evaluate whether the adjuvants can be used to reduce the standard dosage of purgative further. The PubMed/MEDLINE, EMBASE, Cochrane Library, and Web of Science database were searched for randomized controlled trials (RCTs). The primary outcome was the efficacy of bowel preparation, and the secondary outcomes were patients' tolerability and complication rate. The overall quality of evidence was assessed using the GRADEpro guideline development tool. Five RCTs with a total of 1013 patients from Korea were included. The majority of patients were outpatients from different hospitals. The pooled data showed no significant difference in the adequate bowel preparation rate (89.3% versus 89.4%, RR 1, 95% CI 0.95-1.05, *I*^2^ = 47%) as well as in the complication rate (RR for nausea 1.22, 95% CI 0.89-1.65, *I*^2^ = 49%; RR for bloating 0.96, 95% CI 0.73-1.28, *I*^2^ = 0%; RR for vomiting 0.69, 95% CI 0.32-1.50, *I*^2^ = 33%; RR for abdominal pain 1.01, 95% CI 0.61-1.69, *I*^2^ = 0%). But a significantly higher willingness rate was observed in the lower volume (85.1% versus 67.9%, RR 1.25, 95% CI 1.14-1.38, *I*^2^ = 46%). The quality of primary outcome evidence was moderate. The findings of this meta-analysis revealed that 1L PEG-AA may be a viable alternative to 2L PEG-A, with comparable effectiveness, better patient preference, and no statistically significant adverse event occurrence.

## 1. Introduction

Colonoscopy has a significant clinical value in the prevention of colorectal cancer [1]. The importance of bowel preparation quality is highlighted as it is one of the seven key performance measures recommended for colonoscopy by the Quality Committee of the European Society of Gastrointestinal Endoscopy (ESGE) [2]. However, prior to colonoscopy, patients were mainly worried about the colonoscopy itself; the fear of colonoscopy was then followed by bowel preparation [3, 4].

There are various commercially available bowel preparation agents for bowel cleansing. Also, the high volume or low volume polyethylene glycol- (PEG-) based preparations as well as that of non-PEG-based regimens are recommended by guidelines; they require clinical validation for routine bowel preparation [5]. Whereas, due to the lower cost, the 2-4 L PEG-based regimens are the preferred method and are widely used since the introduction of PEG solution in 1980 [6]. The volume of the preparation solution is regarded as the main tolerability and acceptability concern of patients who are preparing for colonoscopy [7].

The issues of poor compliance and the volume of low tolerability of laxatives in bowel preparation have already been considered. The reduction in the volume of PEG in 2L PEG plus ascorbic acid (PEG-Asc) method is a safe and effective bowel preparation method when compared to conventional 4 L PEG method [8, 9]. As a result, the 2L PEG-Asc method is now regarded as a valid alternative to standard PEG and is approved by the United States Food and Drug Administration [10]. However, the dosage of this regimen is still large to ingest. Furthermore, with the prepackaged low-residue diet, a 1L PEG-Asc regimen is shown to be noninferior to low-volume solution in terms of cleansing efficacy [11]. For general bowel preparation, although adjuvants, except simethicone, are not suggested for routine use [5, 12, 13], several recent studies in Korea observed that the additional usage of an adjunct along with the traditional bowel preparation agents can reduce the volume required.

The current review compares the effectiveness of 1L PEG with ascorbic acid plus adjunctive drug preparations (1L PEG-AA, lower volume) with 2L PEG with ascorbic acid regimens (2L PEG-A, low volume) using the meta-analytical techniques. This study is aimed at exploring whether adjunctive drugs can be used for PEG-Asc to reduce the dosage of purgative in a larger pool of patients.

## 2. Methods

### 2.1. Data Sources and Search Strategy

Systematic searches were performed by two independent reviewers (Xin Yuan and Zhixin Zhang) through PubMed/MEDLINE, EMBASE, Cochrane Library, and Web of Science (up to March 10, 2020) with predefined search terms. The search strategy (example of EMBASE in Table S1) was formulated according to the Medical Subject Heading/entrée along with the keywords relating to “colonoscopy,” “polyethylene glycols,” and “1L.” Only full texts published in English were included.

After removing the duplicate studies by the embedded function in the EndNote software, the titles and abstracts were screened, and then, the full texts of the potentially relevant studies were reviewed against the criteria. To avoid the literature omission, we also looked at the references of each literature.

### 2.2. Study Eligibility Criteria

The studies were considered eligible according to the PICOS criteria: (1) participants (P): all adults who received colonoscopy; (2) interventions (I) and comparisons (C): comparison of 1L PEG-AA versus 2L PEG-A before colonoscopy; (3) outcome (O): the primary outcome involves the bowel preparation efficacy as measured by Boston Bowel Preparation Scale (BBPS), and the secondary outcomes include the tolerability and complications associated with the regimen; and (4) study (S): randomized controlled trials (RCTs).

### 2.3. Data Synthesis

Basic characteristics (such as age, sex, comorbidities, indication of colonoscopy, and measures for bowel preparation), quality indicators (the total examination time, adenoma detection rate (ADR), number of experienced endoscopists, cecal intubation rate (CIR)), and outcomes (such as adequate bowel preparation (ABP) rate, total and segments score of BBPS, tolerability, and complications) were extracted from the eligible articles by the two independent investigators (Xin Yuan and Lu Xu). The original data sets are presented in Table S2. Any discrepancies between the investigators were arbitrated and solved by mutual discussion, and reached an agreement with the assistance of a senior investigator (Lei Xu).

### 2.4. Risk of Bias and Validity Assessment

The study quality was evaluated using the Cochrane Collaboration risk of bias tool for RCTs. The tool included seven domains (such as random sequence generation, allocation concealment, blinding of participants and personnel, blinding of outcome assessment, incomplete data, selective reporting, and other bias) of assessment and are classified as low, unclear, or high risk. Due to the inclusion of limited studies, the publication bias was not assessed by funnel plot but done by Egger's test. The validity of the study was assessed by one of the authors (Weihong Wang) by using the GRADEpro GDT approach (https://gdt. http://gradepro.org/app) to summarize quality of the evidence.

### 2.5. Statistical Analysis

The data extracted was consistently collected across all eligible studies, pooled together, and tabulated in a common format in Microsoft Excel. Statistical analyses were performed on the RevMan software (Review Manager Version 5.3) and the STATA software (StataMP Version 14). As our meta-analysis included the continuity variables, i.e., the BBPS score and the dichotomous variables, i.e., willingness to repeat rate, they were separately calculated and displayed using the forest plots with fixed or random effects model. For dichotomous events, the relative risk (RR) along with 95% confidence intervals (CI) was calculated. As the outcomes included were continuous variables, the pooled estimates were calculated as mean differences (MD) as well as 95% CI. Statistical heterogeneity among the trials was assessed by *I*^2^ measure of inconsistency and a *P* value, which was significant if *I*^2^ was >50% or *P* < 0.10. If statistically significant, then study elimination analysis was conducted when possible sources of clinical heterogeneity of a certain study were observed, and a random-effects model was applied. In addition, subgroup analysis was performed when necessary.

## 3. Results

### 3.1. Search Results

**Figure 1:**
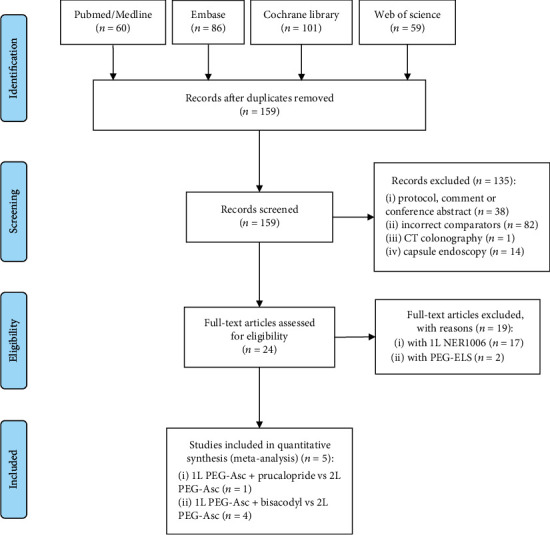
Flow diagram on literature search (PEG-Asc: polyethylene glycol with ascorbic acid; PEG-ELS: polyethylene glycol).

The initial literature research yielded 306 articles, and 147 of these were immediately excluded due to duplications. Next, after reviewing the titles and abstracts, a total of 135 studies were rejected because they were either noncolonoscopy trials, full-texts unavailability, or did not compare bowel preparations under consideration. After that, 24 articles were retrieved for pertinence and full-text reading. From this, a total of nineteen studies were excluded from further evaluation. Seventeen studies were excluded as they compared 1L novel polyethylene glycol-based bowel preparation (NER1006) with 2L PEG-A, and another 2 studies that compared without ascorbic acid were also excluded. Finally, 5 eligible studies were incorporated into the present meta-analysis (Figure 1).

### 3.2. Study Characteristics and Quality

**Figure 2:**
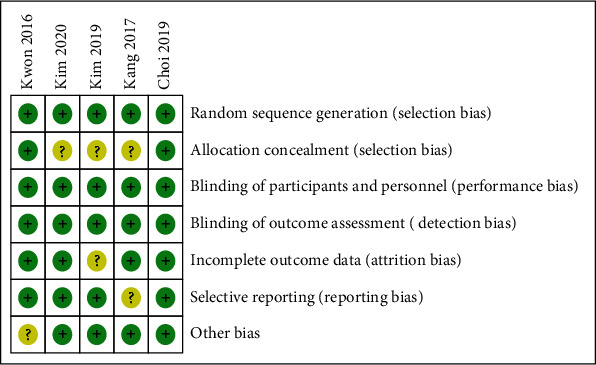
Risk of bias summary.

All the 5 trials (summarized characteristics in Table 1) were carried out in Korea. In the experimental group, four studies performed the bowel preparation with 1L PEG-AA plus bisacodyl, and the remaining used adjunctive drug with prucalopride. All patients received 2L PEG-A split dose bowel preparation in the control group. Of the 5 studies, 4 were single center studies. By seeking the original study protocols that are available in the Clinical Research Information Service (CRIS) and Clinical Trials.gov and by studying thoroughly, the researchers used the same bowel preparation solution (Coolprep®: Sodium Chloride 2.691 g, Potassium Chloride 1.015 g, Anhydrous sodium sulfate 7.5 g, PEG 3350 100 g, ascorbic acid 4.7 g, sodium ascorbate 5.9 g). There was a little evidence of publication bias for primary outcome (*Egger*'*s* test *P* = 0.441), and the risk bias of each study was represented in Figure 2. The quality of primary outcome evidence was moderate (Table S3).

### 3.3. Participant Characteristics

Altogether, 10 bowel preparation arms were analyzed in a total of 1013 patients (503 in the 1L PEG-AA and 510 in the 2L PEG-A). The proportion of male patients, body mass index (BMI), history of previous colonoscopy rate, source of patients, indication of colonoscopy, procedure time, and the diet restrictions were shown to be similar between the two groups in individual trials. CIR and ADR were satisfying in both groups of eligible data (Table 1, Table S4).

### 3.4. Primary Outcome: Efficacy (Overall and Segment Colon)

**Figure 3:**
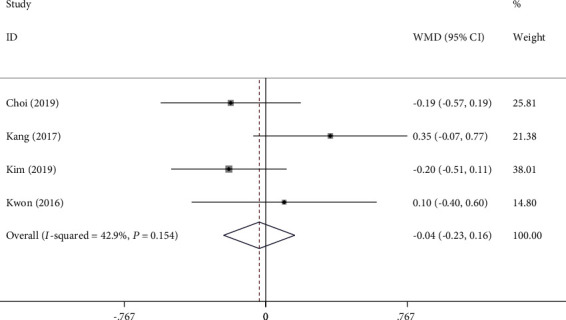
Comparison of forest plot on the adequate bowel preparation rate between 1L PEG-AA and 2L PEG-A (RR: relative risk; CI: confidence interval; 1L PEG-AA refers to 1L polyethylene glycol plus ascorbic acid with adjuvant drug; 2L PEG-A refers to 2L polyethylene glycol plus ascorbic acid).

The quality of bowel cleanliness was reported in 5 trials and was examined with BBPS. In the studies that reported the data on the adequate bowel preparation rate, 1L PEG-AA regimens demonstrated equivalent efficacy of bowel preparation when compared with the 2L PEG-A group (89.3% versus 89.4%; RR 1.00; 95% CI 0.95-1.05; *I*^2^ = 47%) (Figure 3, Table 2).

Based on the data reported on overall and segment colon (right, transverse and left colon), there was no obvious difference in the efficacy between the two bowel cleansing preparations (Fig S1-4, Table 2). However, considerable heterogeneity (*I*^2^ = 86%; *P* < 0.0001) was found in the left colon, and subgroup analysis was performed. The efficacy was slightly lower in 1L PEG-AA regimens (MD -0.11; 95% CI -0.19-0.03; *I*^2^ = 0%; *P* for heterogeneity 0.88) in the subgroup of only extra 0.5 L water intake.

### 3.5. Secondary Outcomes

Other indicators (the total examination time and ADR) related to intestinal cleanliness efficacy were available. No significant trend towards more adenomas (43.7% vs. 43.2%; RR 1.01; 95% CI 0.86-1.18; *I*^2^ = 0%) was detected in 2L PEG-A than 1L PEG-AA, showing no much difference in the total examination time (SMD -0.86; 95% CI -1.88-0.15; *I*^2^ = 27%) (Table 2).

**Figure 4:**
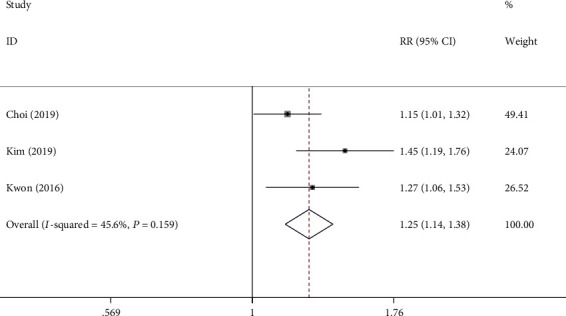
Comparison of forest plot on the willingness of 1L PEG-AA versus 2L PEG-A (RR: relative risk; CI: confidence interval; 1L PEG-AA refers to 1L polyethylene glycol plus ascorbic acid with adjuvant drug; 2L PEG-A refers to 2L polyethylene glycol plus ascorbic acid).

Data on the number of patients with preference were available in 3 out of 5 treatment arms. Overall, 234/275 (85.1%) patients in the 1L PEG-AA group vs. 190/280 (67.9%) in the 2L PEG-A group showed willingness to repeat the same regimens with a pooled RR of 1.25 (95% CI 1.14-1.38; *I*^2^ = 46%) (Figure 4, Table 3). Two studies reported the completion rates, which were similar. The pooled RR was 1.03 (95% CI 0.95-1.07; *I*^2^ = 8%) and showed no statistically significant differences with low heterogeneity.

All studies reported few similar complications (such as nausea, abdominal fullness/bloating, vomiting, and abdominal pain), which were extracted and analyzed. However, the latest study combined nausea and vomiting, so separate data could not be extracted. Nausea with 14.5% (118/815) and bloating with 14.8% (150/1013) were the more frequent complications than the others, while the incidence showed no statistical significance in the two groups (respectively, RR for nausea 1.22 (95% CI 0.89-1.65), *I*^2^ = 49%; RR for bloating 0.96 (95% CI 0.73-1.28), *I*^2^ = 0%). Vomiting was less frequently reported, and no statistical significance was observed, in the control group (1.2% vs. 1.8%; RR 0.69; 95% CI 0.32-1.50; *I*^2^ = 33%). No differences were observed for abdominal pain in the two groups (2.7% vs. 2.7%; RR 1.01; 95% CI 0.61-1.69; *I*^2^ = 0%) (Fig S5-8, Table 3).

## 4. Discussion

To the best of our knowledge, this is the first meta-analysis study that pooled eligible individual data from RCTs comparing 1L PEG-AA with 2L PEG-Asc alone and also defined the bowel preparation quality with BBPS scoring as an outcome. According to our study, the main finding indicated that the efficiency and safety of 1L PEG-AA was not inferior to 2L PEG-A but observed more willingness for patients to repeat the 1L PEG-AA. Improving the willingness of patients for bowel preparation is important in improving the colonoscopy rate and thus will benefit to prevent the colorectal cancer.

When describing the preparation quality, the BBPS is deemed to be the most thoroughly validated scale [14] and thought to be used in a clinical setting for all the published scales that displayed limitations [15]. Furthermore, when more than one grader participates in the rating for each colonoscopy, then a dichotomized endpoint might be recommended [16]. Therefore, we used the adequate bowel preparation rate as the primary outcome. All the endoscopists in the current study were well trained to eliminate bias and used BBPS to rate based on their own experience. What is more, comprehensive consideration of total and segmental colon scores also remained a good way to confirm the results of comparing the differences in bowel preparation.

The included studies have individually evaluated the effectiveness of adjuvant drugs in reducing the intestinal preparation solutions, one with prucalopride [17] and the remaining four with bisacodyl [18–21]. Our review highlighted the feasibility of the use of combination for bowel preparation after summarizing in 1013 patients with the results (either primary or secondary outcomes). The results showed no significant disadvantages in the lower-volume group. While for the cleanliness of the left colon, Kang et al. [19] and Kim et al. [21] found that the recto-sigmoid colon was more statistically effective in the 1L PEG-AA group (2.57 versus 2.26). In addition to the disparities in statistical findings, the sources of heterogeneity within the study were explored and observed that it might be due to the addition of different amounts of water, and an additional 1L of water given as the last dose when compared to 0.5 L in other studies (Table 1). It would be very interesting to evaluate whether additional water not with laxative intake can affect the intestinal cleanliness. Another explanation might be the difference in the dosage and type of adjuvant use.

Furthermore, many patient-related factors influence the bowel preparation, inevitably leading to heterogeneity. As several previous studies indicated, the predictors such as age, sex, BMI, previous history of colonoscopy, dietary restrictions, distinction in the brand of medication, indications of colonscopy, and interval time before the procedure showed association with colon cleansing [22, 23]. Almost all the studies included in this meta-analysis were internally consistent in these respects in order to eliminate the influence of these confounding factors on the results. Nevertheless, in Kim's trial, a statistically significant difference was observed in the mean age of patients [20]. Our study results showed that patients in the 2L group (48.14 vs. 52.88) were younger, which was under the common cut-off, and the results ultimately showed no significant difference between the two groups [22].

Nevertheless, according to the standard of intestinal preparation, we believe that a BBPS score of ≥2 for all segments and/or a total score ≥ 6 are sufficient [24]. Although the guideline pointed that a minimum standard of ≥90% for adequate bowel preparation as assessed using the validated scales, only a minority of centers could achieve, and the literatures indicated that the inadequate bowel preparation rate was between 5% ~30% at present [2, 25]. The ABP rate in this study with pooled rate of 89% ranged from 83% to 96%, all of which were at the mid-to-upper level in clinical practice. What is more, ADR and CIR were both above the minimum standard (ADR ≥ 20%, CIR ≥ 90%) for colonoscopy in each trials according to current guideline (Table S4) [2]. Perhaps as long as the laxative is taken at a certain dose, other aspects of concern rather than the dose may play a more important role in intestinal preparation.

Another marked advantage of this protocol is the willingness of patients to repeat, enrollment of sufficient number of patients, and preference for lower-dose regimens is visibly demonstrated [26]. The lower-dose regimen that substitutes an adjuvant for large quantities of solution the day before colonoscopy, particularly the additional suppository using on the day of colonoscopy, can reduce the disturbance on sleep [20]. Secondly, it reduces the consumption of these unpalatable liquids, which can reduce the psychological burden on patients. Patients' willingness should be highly considered in spite of the similar completion rates of the patients, and high-prep approach for all should be discouraged [27–29].

Patient complications mainly focused on the most common adverse events including nausea, vomiting, abdominal pain, and diarrhea [30]. The results of this study showed no statistical differences, while only a conservative estimation can be done due to limited sample size in this part. Based on the current available literature, our results supported the use of additional adjuvants solely with PEG-Asc solution [17–21]. According to a hypothesis, the cathartic effect of bisacodyl and prucalopride might be due to its cathartic or prokinetic mechanism, which in turn changes the motor patterns in the colon and promotes the intestinal movement [31, 32].

At present, the adequate bowel preparation rate has been greatly improved clinically [29], but the experience of taking too many laxatives still remained a prominent problem. The role of adjuvants in the lower-volume PEG-Asc has been unclear, and the current guideline states that the use of prokinetic agents is not routinely suggested [5]. Thus, they are not widely used in daily clinical practice. Despite the finite published data in some studies, this review provides evidence that no obvious disadvantages with regard to effectiveness, and safety was presented with 1L PEG-AA regimens in outpatients.

Our analysis included several patients as well as found acceptable heterogeneity in most of the comparisons on major outcomes. Several limitations in this study should be acknowledged. Firstly, less number of studies was included, and all the studies were sourced from a single country; therefore, the generalizability of the results is questionable and needs more studies to verify the results. Secondly, not all details reported in this study were analyzed due to limited information on the palatability and general discomfort, which might be relevant but not clinically important. Thirdly, almost all the included populations were outpatients, and in consideration of these limitations, our study results should be applied with caution. According to the previous study, the outpatient situation services remained a low risk factor, indicating that 1L PEG-AA might be more suitable for low risk population [33]. Finally, the auxiliary drugs used were only bisacodyl and prucalopride, and further study is warranted to explore the effectiveness of combining other drugs such as probiotics, mosapride, magnesium citrate, and castor oil with 1L PEG-Asc.

## 5. Conclusion

In conclusion, lower-volume PEG with adjuvants might be considered as an alternative to the conventional low-dose (2L) PEG for bowel preparation prior to colonoscopy. Future efforts should focus on more approaches to decrease the intake of laxatives. This study should preferably include approximately 1863 patients (power = 80%, alpha = 0.05, with 10% risk reduction).

## Figures and Tables

**Table 1:** Baseline characteristics of included randomized controlled trials.

Study, year (country)	Source of patients	Experimental groups	1L, *n*	2L, *n*	Use of adjuvant	Time of first and last dose	Procedure time	Diet	Outcomes	Type of study
Kwon et al. [18], 2016 (Korea)	Outpatient	FD: Bis+0.5 L water LD: 1 L PEG − Asc + 0.5 L water	91	96	20 mg bisacodyl	FD: 8 pm day-prior LD: 6 am day-of	≥3 h interval before	3-day low residual	BBPS	Multicenter, single-blind, RCT
Kang et al. [19], 2017 (Korea)	Outpatient	FD: Bis LD: 1 L PEG − Asc + 1 L water	100	100	10 mg bisacodyl	FD: 9 pm day-prior LD: ≥4 h prior	9 am-13 pm	3-day low residual	BBPS, ABPS	Single-center, single-blind, RCT
Choi et al. [17], 2019 (Korea)	Outpatient	FD: Pru + 0.5 L water LD: 1 L PEG − Asc + 0.5 L water	130	130	2 mg prucalopride	FD: 7 pm day-prior LD: ≥5 h prior	9 am-13 pm	3-day low residual	BBPS, ABPS	Single-center, single-blind, RCT
Kim et al. [20], 2019 (Korea)	Outpatient	FD: — LD: Bis + 1 L PEG − Asc + 0.5 L water	83	85	20 mg bisacodyl (suppository)	FD: — LD: 5 am day-of	8 : 30 am-12 pm	1-day clear liquid	BBPS	Single-center, single-blind, RCT
Kim et al. [21], 2020 (Korea)	NA	FD: Bis LD: 1 L PEG − Asc + 1 L water	99	99	10 mg bisacodyl	FD: 9 pm day-prior LD: ≥5 h prior	9 am-17 pm	3-day low residual	BBPS	Single-center, single-blind, RCT

ABPS: Aronchick Bowel Preparation Scale; BBPS: Boston Bowel Preparation Scale; Bis: bisacodyl; FD: first dose; LD: last dose; PEG-Asc: polyethylene glycol plus ascorbic acid; Pru: prucalopride; RCT: randomized controlled trial; 1 L: 1 L polyethylene glycol plus ascorbic acid with adjuvant drug; 2 L: 2 L polyethylene glycol plus ascorbic acid.

**Table 2:** Analysis of results of primary outcome and impact on the procedure in terms of efficacy of cleansing.

Efficacy	No. of studies	*n*	MD/RR	95% CI	*P*	*I* ^2^	*P* value for heterogeneity
Adequate bowel preparation rate	4	845	1.00	0.95~1.05	0.87	47%	0.13
BBPS							
Right colon	5	1013	-0.07	-0.15~0.01	0.13	16%	0.31
Transverse colon	5	1013	0.03	-0.04~0.10	0.39	0%	0.57
Left colon	5	1013	0.05	-0.14~0.24	0.62	86%	<0.001
Total	5	1013	0.01	-0.17~0.18	0.83	36%	0.18
Total examination time	4	826	-0.86	-1.88~0.15	0.14	27%	0.25
ADR	4	826	1.01	0.86~1.18	0.91	0%	0.60

*I* ^2^ indicates heterogeneity. ADR: adenoma detection rate; BBPS: Boston Bowel Preparation Scale; CI: confidence interval; MD: mean difference; *P* for each MD/RR 95% Cl analysis; RR: relative risk.

**Table 3:** Analysis of results for secondary outcomes in terms of patient experience.

Tolerability and safety	No. of studies	*n*	RR	95% CI	*P*	*I* ^2^	*P* value for heterogeneity
Tolerability							
Willingness to repeat	3	555	1.25	1.14~1.38	<0.001	46%	0.16
Completion rate	2	447	1.03	0.98~1.07	0.21	8%	0.30
Safety							
Nausea	4	815	1.22	0.89~1.65	0.21	49%	0.12
Vomiting	4	815	0.69	0.32~1.50	0.35	33%	0.21
Abdominal pain	5	1013	1.01	0.61~1.69	0.06	0%	0.72
Abdominal fullness/bloating	5	1013	0.96	0.73~1.28	0.80	0%	0.63

*I* ^2^ indicates heterogeneity. CI: confidence interval; *P* for each MD/RR 95% Cl analysis; RR: relative risk.

## Supplementary Materials

Supplementary Materials Table S1: EMBASE search strategy. Table S2: original data sets. Table S3: GRADE summary of findings. Table S4: baseline characteristics of patients. Figure S1-S4: comparison of forest plot on the BBPS total and segment score between 1L PEG-AA and 2L PEG-A. Figure S5-S8: comparison of forest plot on the adverse events rate between 1L PEG-AA versus 2L PEG-A. The weighted mean difference (WMD) and 95% confidence intervals (CI) for efficacy of the colon according to BBPS score. The relative risk (RR) and 95% CI for adverse events. 1L PEG-AA refers to 1L polyethylene glycol plus ascorbic acid with adjuvant drug; 2L PEG-A refers to 2L polyethylene glycol plus ascorbic acid.
