# Supplementary material for: Comparison of 1L Adjuvant Auxiliary Preparations with 2L Solely Polyethylene Glycol plus Ascorbic Acid Regime for Bowel Cleaning: A Meta-analysis of Randomized, Controlled Trials
Source: Biomed Res Int. 2021 Feb 18;2021:6638858. doi: 10.1155/2021/6638858 (PMC7910058; doi:10.1155/2021/6638858)
Supplement: Supplementary Materials — Table S1: EMBASE search strategy. Table S2: original data sets. Table S3: GRADE summary of findings. Table S4: baseline characteristics of patients. Figure S1-S4: comparison of forest plot on the BBPS total and segment score between 1L PEG-AA and 2L PEG-A. Figure S5-S8: comparison of forest plot on the adverse events rate between 1L PEG-AA versus 2L PEG-A. The weighted mean difference (WMD) and 95% confidence intervals (CI) for efficacy of the colon according to BBPS score. The relative risk (RR) and 95% CI for adverse events. 1L PEG-AA refers to 1L polyethylene glycol plus ascorbic acid with adjuvant drug; 2L PEG-A refers to 2L polyethylene glycol plus ascorbic acid. [file 6638858.f1.doc]

**Comparison of 1L Adjuvant Auxiliary Preparations with 2L Solely Polyethylene Glycol plus Ascorbic Acid Regime for Bowel Cleaning: A Meta-analysis of Randomized, Controlled Trials**

**Xin Yuan,^1,2^ Zhixin Zhang,^1,2^ Jiarong Xie,^2^ Yu Zhang,^2^ Lu Xu,^3^ Weihong Wang,^2^ and Lei Xu^2^**

*^1^ College of Medicine, Ningbo University, Zhejiang, China*

*^2^* *Department of Gastroenterology, Ningbo First Hospital, Zhejiang, China*

*^3^* *Clinical Department for Intensive Care, Ningbo No.2 Hospital, Zhejiang, China*

Correspondence should be addressed to Lei Xu; [xulei22@163.com](mailto:xulei22@163.com)

**Supplementary figure legends**

**Fig S1** Comparison of forest plot on the BBPS total score between 1L-PEG-AA and 2L-PEG-A. The weighted mean difference (WMD) and 95% confidence intervals (CI) for efficacy of total colon according to BBPS score. 1L-PEG-AA refers to 1L polyethylene glycol plus ascorbic acid with adjuvant drug; 2L-PEG-A refers to 2L polyethylene glycol plus ascorbic acid.

**Fig S2** Comparison of forest plot on the right colon BBPS score between 1L-PEG-AA and 2L-PEG-A. The weighted mean difference (WMD) and 95% confidence intervals (CI) for efficacy of right colon according to BBPS score. 1L-PEG-AA refers to 1L polyethylene glycol plus ascorbic acid with adjuvant drug; 2L-PEG-A refers to 2L polyethylene glycol plus ascorbic acid.

**Fig S3** Comparison of forest plot on the transverse colon BBPS score between 1L-PEG-AA and 2L-PEG-A. The WMD (95%CI) for efficacy of transverse colon according to BBPS. 1L-PEG-AA refers to 1L polyethylene glycol plus ascorbic acid with adjuvant drug; 2L-PEG-A refers to 2L polyethylene glycol plus ascorbic acid.

**Fig S4** Comparison of forest plot on the left colon BBPS score between 1L-PEG-AA and 2L-PEG-A. The WMD (95%CI) for efficacy of left colon according to BBPS in subgroup based on the additional water consumption of 0.5L and 1L.1L-PEG-AA refers to 1L polyethylene glycol plus ascorbic acid with adjuvant drug; 2L-PEG-A refers to 2L polyethylene glycol plus ascorbic acid.

**Fig S5** Comparison of forest plot on the nausea rate between 1L-PEG-AA versus 2L-PEG-A. The relative risk (RR) and 95%CI for nausea.1L-PEG-AA refers to 1L polyethylene glycol plus ascorbic acid with adjuvant drug; 2L-PEG-A refers to 2L polyethylene glycol plus ascorbic acid.

**Fig S6** Comparison of forest plot on the vomiting rate between 1L-PEG-AA and 2L-PEG-A. The RR (95%CI) for vomiting. 1L-PEG-AA refers to 1L polyethylene glycol plus ascorbic acid with adjuvant drug; 2L-PEG-A refers to 2L polyethylene glycol plus ascorbic acid.

**Fig S7** Comparison of forest plot on the abdominal pain rate between 1L-PEG-AA and 2L-PEG-A. The RR (95%CI) for abdominal pain.1L-PEG-AA refers to 1L polyethylene glycol plus ascorbic acid with adjuvant drug; 2L-PEG-A refers to 2L polyethylene glycol plus ascorbic acid.

**Fig S8** Comparison of forest plot on the abdominal fullness rate between 1L-PEG-AA versus 2L-PEG-A. The RR (95%CI) for abdominal fullness. 1L-PEG-AA refers to 1L polyethylene glycol plus ascorbic acid with adjuvant drug; 2L-PEG-A refers to 2L polyethylene glycol plus ascorbic acid.

**Table S1** EMBASE search strategy

| **NO.** | **Query** | **Results** |
| --- | --- | --- |
| **1** | **'colonoscopy'/exp OR** **colonoscop*OR coloscop*** | **85312** |
| **2** | **'macrogol'/exp OR 'macrogol':ab,ti OR 'macrogols' :ab,ti OR 'polyoxyethylene':ab,ti OR 'polyoxyethylenes':ab,ti OR 'polyglycol':ab,ti OR 'polyglycols':ab,ti OR 'polyethylene glycol':ab,ti OR 'polyethylene glycols':ab,ti OR 'carbowax':ab,ti** | **67037** |
| **3** | **'1l':ab,ti OR '1liter' :ab,ti OR '1litre':ab,ti OR '1-l':ab,ti** | **18077** |
| **4** | **#1 AND #2 AND #3** | **119** |
| **5** | **'crossover procedure':de OR 'double-blind procedure':de OR 'randomized controlled trial':de OR 'single-blind procedure':de OR random*:de,ab,ti OR factorial*:de,ab,ti OR crossover*:de,ab,ti OR ((cross NEXT/1 over*):de,ab,ti) OR placebo*:de,ab,ti OR ((doubl* NEAR/1 blind*):de,ab,ti) OR ((singl* NEAR/1 blind*):de,ab,ti) OR assign*:de,ab,ti OR allocat*:de,ab,ti OR volunteer*:de,ab,ti** | **2532521** |
| **6** | **#4 AND #5** | **86** |

**Table S2** Original data sets

| **Study**  **year** | **ABP, n(%)** | |  | **Total,**  **mean±SD** | |  | **right colon,**  **mean±SD** | |  | **transverse colon, mean±SD** | |  | **left colon,**  **mean±SD** | |
| --- | --- | --- | --- | --- | --- | --- | --- | --- | --- | --- | --- | --- | --- | --- |
|  | **1L** | **2L** |  | **1L** | **2L** |  | **1L** | **2L** |  | **1L** | **2L** |  | **1L** | **2L** |
| **Kwon**  **2016** | 87 (96) | 84 (88) |  | 7.70±1.70 | 7.60±1.80 |  | 2.50±0.60 | 2.40±0.70 |  | 2.60±0.50 | 2.60±0.60 |  | 2.60±0.50 | 2.70±0.50 |
| **Kang**  **2017** | 95  (95) | 96  (96) |  | 6.92±1.63 | 6.57±1.37 |  | 2.02±0.81 | 2.19±0.66 |  | 2.37±0.70 | 2.22±0.57 |  | 2.57±0.72 | 2.16±0.66 |
| **Choi**  **2019** | 108 (83) | 115 (89) |  | 6.93±1.65 | 7.12±1.47 |  | 2.02±0.63 | 2.15±0.65 |  | 2.31±0.71 | 2.36±0.61 |  | 2.56±0.61 | 2.65±0.55 |
| **Kim**  **2019** | NA | NA |  | 7.73±1.01 | 7.93±1.06 |  | 2.34±0.52 | 2.42±0.52 |  | 2.83±0.38 | 2.80±0.40 |  | 2.57±0.50 | 2.71±0.46 |
| **Kim**  **2020** | 85 (86) | 85 (86) |  | 7.03±1.51 | 6.84±1.39 |  | 2.07±0.81 | 2.11±0.77 |  | 2.40±0.70 | 2.35±0.58 |  | 2.56±0.69 | 2.36±0.65 |

ABP, adequate bowel preparation; SD, standard deviation; 1L refers to 1L polyethylene glycol plus ascorbic acid with adjuvant drug; 2L refers to 2L polyethylene glycol plus ascorbic acid.

1. Original data sets of BBPS

| **Study**  **year** | **Willingness, n(%)** | |  | **Nausea, n(%)** | |  | **Vomiting, n(%)** | |  | **Abdominal pain, n(%)** | |  | **Bloating, n(%)** | |
| --- | --- | --- | --- | --- | --- | --- | --- | --- | --- | --- | --- | --- | --- | --- |
|  | **1L** | **2L** |  | **1L** | **2L** |  | **1L** | **2L** |  | **1L** | **2L** |  | **1L** | **2L** |
| **Kwon**  **2016** | 61  **(84.7)** | 52  **(66.7)** |  | **17**  **(18.7)** | **9**  **(9.4)** |  | **1**  **(1.1)** | **6**  **(6.3)** |  | **6**  **(6.6)** | **3**  **(3.1)** |  | **12 (13.2)** | **10 (10.4)** |
| **Kang**  **2017** | NA | NA |  | **6**  **(6)** | **2**  **(2)** |  | **2**  **(2)** | **0**  **(0)** |  | **1**  **(1)** | **0**  **(0)** |  | **9**  **(9)** | **6**  **(6)** |
| **Choi**  **2019** | 107  **(82.3)** | 93  **(71.5)** |  | **5**  **(3.8)** | **10**  **(7.7)** |  | **5**  **(3.8)** | **4**  **(3.1)** |  | **5**  **(3.8)** | **7**  **(5.4)** |  | **7**  **(5.4)** | **12 (9.2)** |
| **Kim**  **2019** | 66  **(90.4)** | 45  **(62.5)** |  | **36**  **(43.4)** | **33 (38.8)** |  | **2**  **(2.4)** | **5**  **(5.9)** |  | **6**  **(7.2)** | **7**  **(8.2)** |  | **28 (33.7)** | **29 (34.1)** |
| **Kim**  **2020** | **NA** | **NA** |  | **NA** | **NA** |  | **NA** | **NA** |  | **9**  **(9.2)** | **10**  **(10.0)** |  | **17**  **(17.2)** | **20**  **(20.3)** |

**NA, not available; 1L refers to 1L polyethylene glycol plus ascorbic acid with adjuvant drug; 2L refers to 2L polyethylene glycol plus ascorbic acid.**

1. Original data sets of willingness and adverse events

**Table S3** GRADE summary of findings

1. some studies do not apply obvious allocation concealment

| **1L-PEG-AA compared to 2L-PEG-A for bowel preparation** | | | | | | |
| --- | --- | --- | --- | --- | --- | --- |
| **Patient or population**: colonoscopy  **Setting**: bowel preparation  **Intervention**: 1L-PEG-AA  **Comparison**: 2L-PEG-A | | | | | | |
| Outcomes | **Anticipated absolute effects^*^**(95% CI) | | Relative effect (95% CI) | № of participants  (studies) | Certainty of the evidence (GRADE) | Comments |
|  | **Risk with 2L-PEG-A** | **Risk with 1L-PEG-AA** |  |  |  |  |
| Adequate bowel preparation | 894 per 1,000 | **893 per 1,000** (731 to 947) | **RR 1.00** (0.86 to 1.24) | 845 (4 RCTs) | ⨁⨁⨁◯ MODERATE ^a^ |  |
| Adenoma detection rate | 432 per 1,000 | **437 per 1,000** (358 to 516) | **RR 1.01** (0.86 to 1.18) | 826 (4 RCTs) | ⨁⨁◯◯ LOW ^a,b^ |  |
| willingness | 679 per 1,000 | **848 per 1,000** (774 to 936) | **RR 1.25** (1.14 to 1.38) | 555 (3 RCTs) | ⨁⨁⨁◯ MODERATE ^a^ |  |
| Nausea | 131 per 1,000 | **160 per 1,000** (117 to 217) | **RR 1.22** (0.89 to 1.65) | 815 (4 RCTs) | ⨁⨁◯◯ LOW ^a,b^ |  |
| Vomiting | 36 per 1,000 | **25 per 1,000** (12 to 55) | **RR 0.69** (0.32 to 1.50) | 815 (4 RCTs) | ⨁⨁◯◯ LOW ^a,b^ |  |
| abdominal pain | 41 per 1,000 | **45 per 1,000** (24 to 84) | **RR 1.08** (0.57 to 2.04) | 1013 (5 RCTs) | ⨁⨁◯◯ LOW ^a,b^ |  |
| abdominal fullness | 139 per 1,000 | **139 per 1,000** (100 to 193) | **RR 1.00** (0.72 to 1.39) | 1013 (5 RCTs) | ⨁⨁◯◯ LOW ^a,b^ |  |
| ***The risk in the intervention group** (and its 95% confidence interval) is based on the assumed risk in the comparison group and the **relative effect** of the intervention (and its 95% CI).  **CI:** Confidence interval; **RR:** Risk ratio | | | | | | |
| **GRADE Working Group grades of evidence** **High certainty:** We are very confident that the true effect lies close to that of the estimate of the effect **Moderate certainty:** We are moderately confident in the effect estimate: The true effect is likely to be close to the estimate of the effect, but there is a possibility that it is substantially different **Low certainty:** Our confidence in the effect estimate is limited: The true effect may be substantially different from the estimate of the effect **Very low certainty:** We have very little confidence in the effect estimate: The true effect is likely to be substantially different from the estimate of effect | | | | | | |

1. the actual sample is smaller than optimal information sample

**Table S4** Baseline characteristics of patients

| **Study** | **Kwon 2016** | |  | **Kang 2017** | |  | **Choi 2019** | |  | **Kim 2019** | |  | **Kim 2020** | |
| --- | --- | --- | --- | --- | --- | --- | --- | --- | --- | --- | --- | --- | --- | --- |
|  | **1L** | **2L** |  | **1L** | **2L** |  | **1L** | **2L** |  | **1L** | **2L** |  | **1L** | **2L** |
| **Age, mean±SD, y** | 59.6±12.5 | 56.0±7.7 |  | 54.4±14.4 | 56.8±14.0 |  | 55.3±14.4 | 58.5±13.7 |  | 52.9±11.2 | 48.1±12.2 |  | 54.4±12.9 | 58.1±13.2 |
| **Male, %** | 52.7 | 53.1 |  | 52 | 54 |  | 51.5 | 56.2 |  | 41.0 | 45.9 |  |  |  |
| **BMI, mean±SD, kg/m^2^** | NA | NA |  | 23.6±4.1 | 23.4±3.3 |  | 23.5±2.6 | 23.7±2.6 |  | 23.6±3.3 | 23.2±3.5 |  | 23.6±3.4 | 23.1±3.2 |
| **Comorbidities, %** | NA | NA |  |  |  |  |  |  |  |  |  |  |  |  |
| Hypertension | - | - |  | 25 | 31 |  | 25.4 | 20.0 |  | 21.7 | 18.8 |  | 28.3 | 31.3 |
| Diabetes mellitus | - | - |  | 6 | 7 |  | 3.8 | 5.4 |  | 9.6 | 8.2 |  | 12.1 | 13.1 |
| Cardiovascular disease | - | - |  | 6 | 10 |  | 7.7 | 4.6 |  | 1.2 | 0 |  | 11.1 | 10.1 |
| Others | - | - |  | 29 | 18 |  | 9.2 | 13.8 |  | NA | NA |  | 29.3 | 33.3 |
| **Previous colonoscopy, %** | 79.1 | 81.3 |  | NA | NA |  | NA | NA |  | 62.7 | 61.2 |  | NA | NA |
| **Indication of colonoscopy, %** | NA | NA |  |  |  |  |  |  |  |  |  |  |  |  |
| Screening | - | - |  | 23 | 30 |  | 43.1 | 40.8 |  | 26.5 | 25.5 |  | 50.5 | 56.1 |
| History of colon polyp | - | - |  | 26 | 34 |  | 28.5 | 23.8 |  | 42.2 | 40.0 |  | 24.2 | 22.2 |
| Abdominal pain | - | - |  | 26 | 17 |  | 8.5 | 10.8 |  | 9.6 | 12.9 |  | 10.1 | 5.1 |
| Overt bleeding | - | - |  | 4 | 4 |  | 8.5 | 7.7 |  | NA | NA |  | 1.0 | 1.0 |
| Anemia | - | - |  | 2 | 1 |  | 3.8 | 3.1 |  | 1.2 | 0 |  | 2.0 | 2.0 |
| Loose stool | - | - |  | 9 | 5 |  | 2.3 | 6.2 |  | 14.5 | 4.7 |  | 2.0 | 4.0 |
| Weight loss | - | - |  | NA | NA |  | 1.5 | 3.8 |  | NA | NA |  | NA | NA |
| Occult blood in stool | - | - |  | 5 | 3 |  | 3.8 | 3.8 |  | 10.8 | 15.3 |  | 2.0 | 1.0 |
| **Previous abdominal surgery, %** | 0 | 0 |  | 21 | 21 |  | 20.8 | 22.3 |  | NA | NA |  | NA | NA |
| **Constipation, %** | NA | NA |  | NA | NA |  | 13.1 | 8.5 |  | 18.1 | 14.1 |  | 16.2 | 14.1 |
| **Endoscopist**^*^**, n** | 2 | |  | 2 | |  | 1 | |  | 2 | |  | 3 | |
| **Cecal intubation, %** | NA | NA |  | 100 | 100 |  | 100 | 100 |  | 100 | 100 |  | 100 | 100 |
| **ADR, %** | NA | NA |  | 45 | 45 |  | 45.4 | 38.5 |  | 37.4 | 42.4 |  | 45.5 | 48.5 |

ADR, adenomas detection rate; BMI, body mass index; CIR, cecal intubation rate; SD, standard deviation; NA, not available; 1L refers to 1L polyethylene glycol plus ascorbic acid with adjuvant drug; 2L refers to 2L polyethylene glycol plus ascorbic acid.

* endoscopists were all experienced.


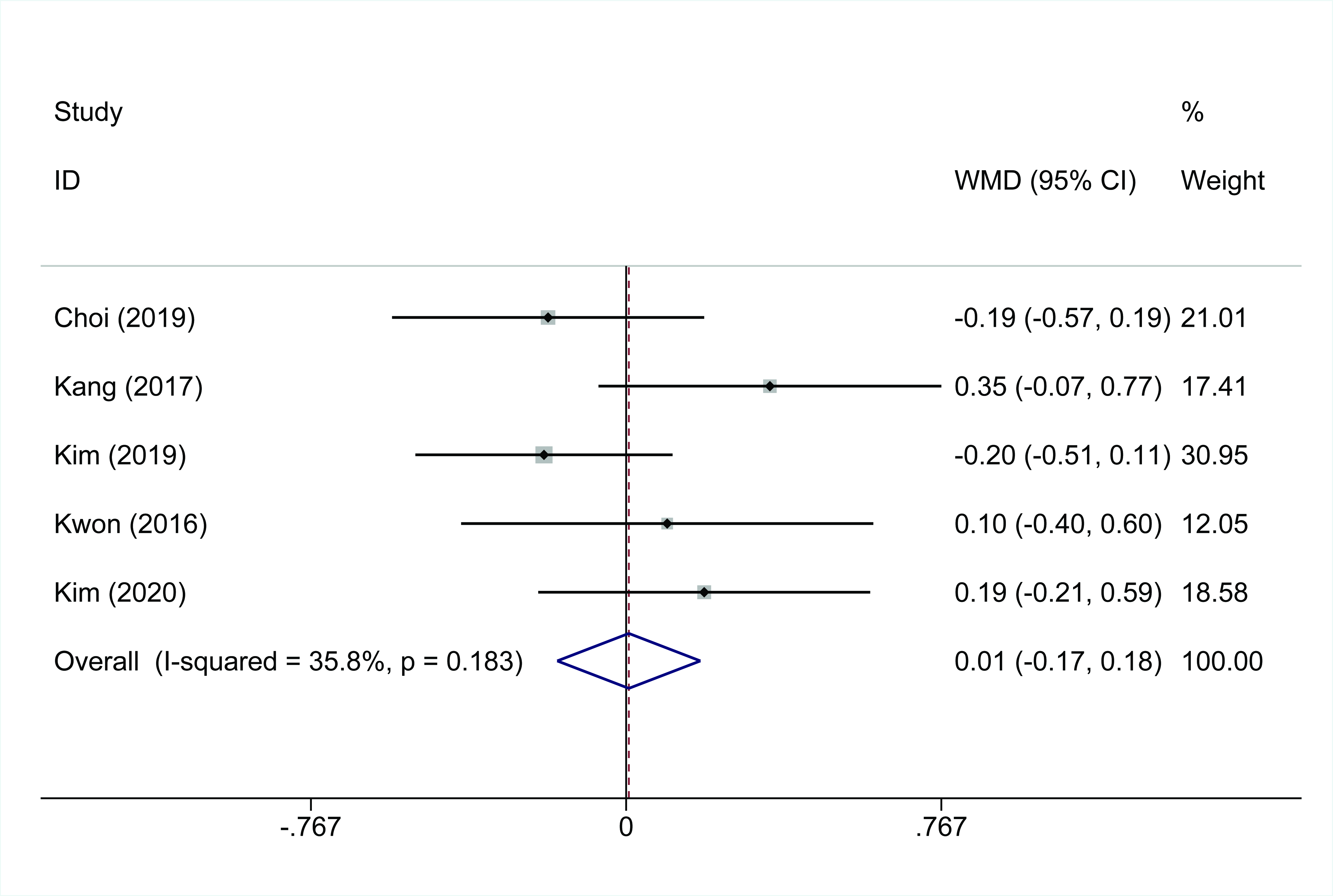
**Fig S1**


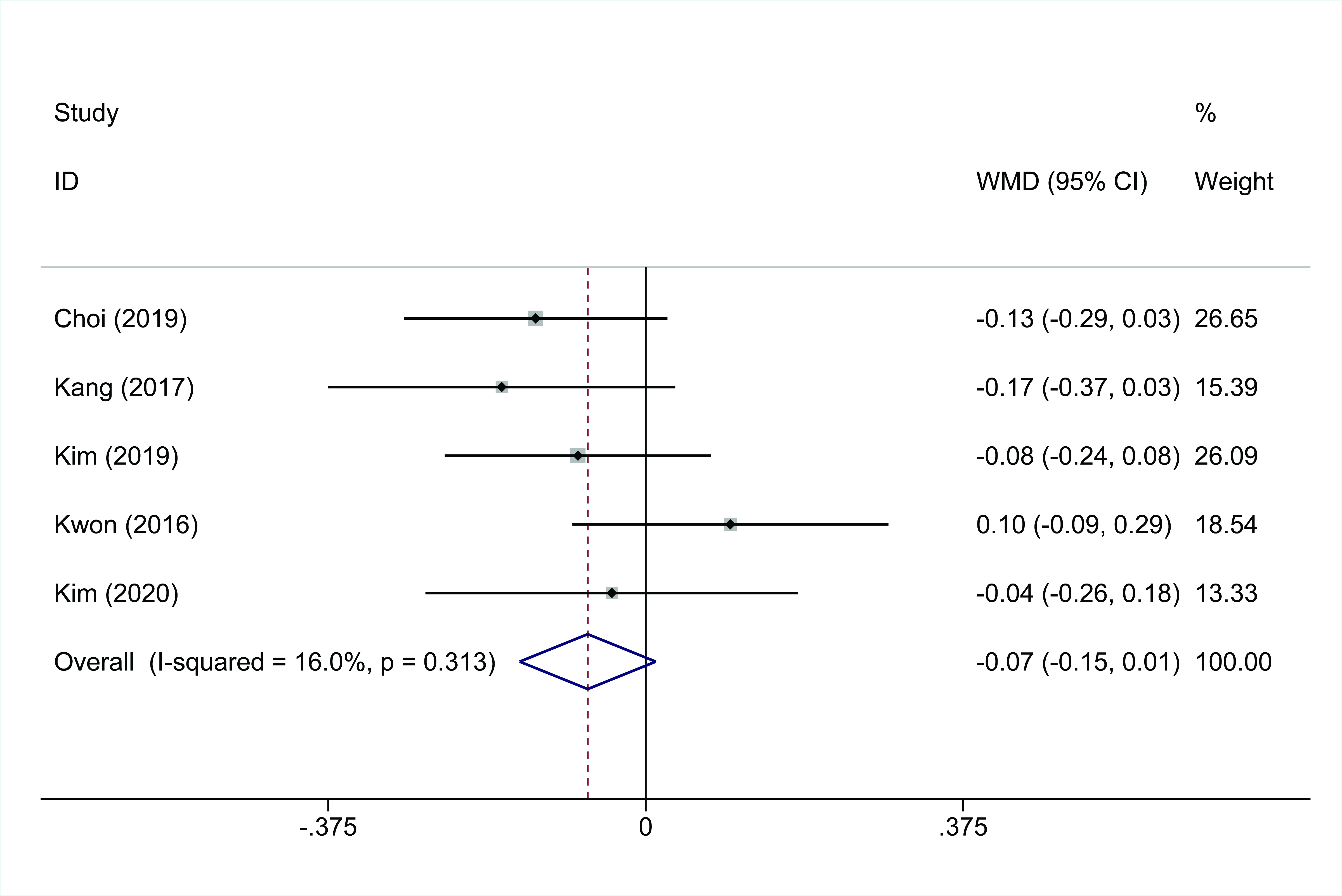
**Fig S2**


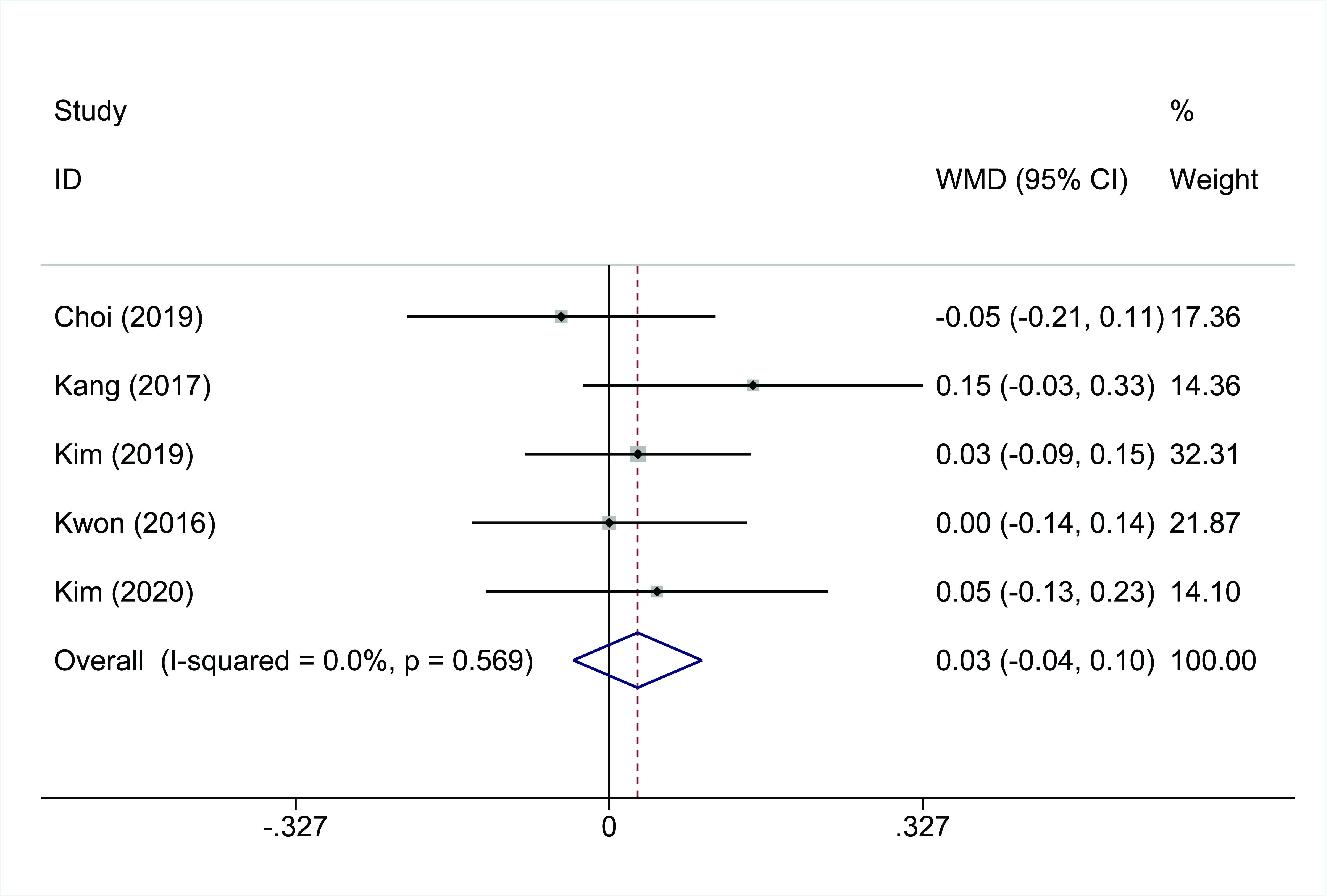
**Fig S3**


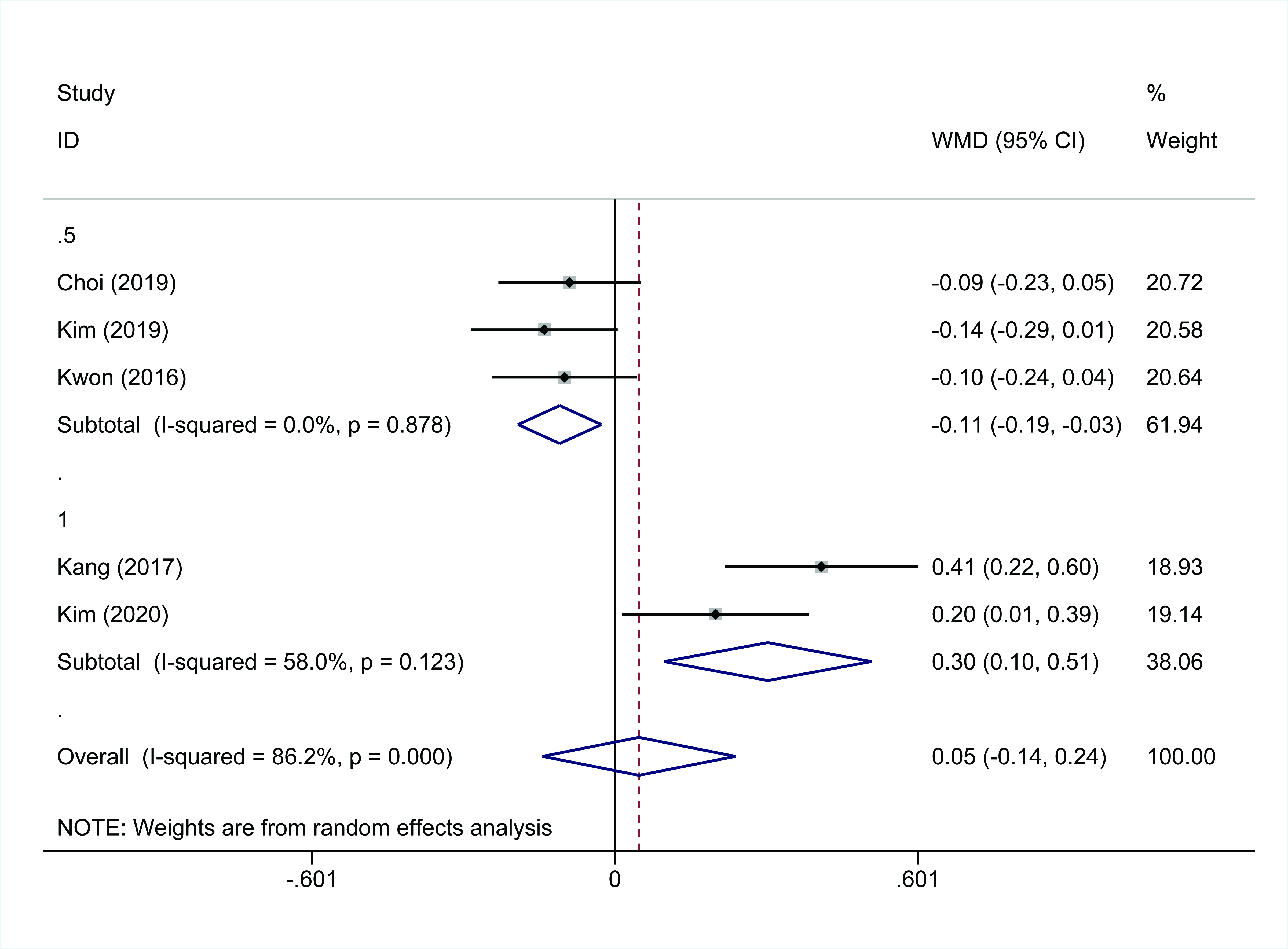
**Fig S4**


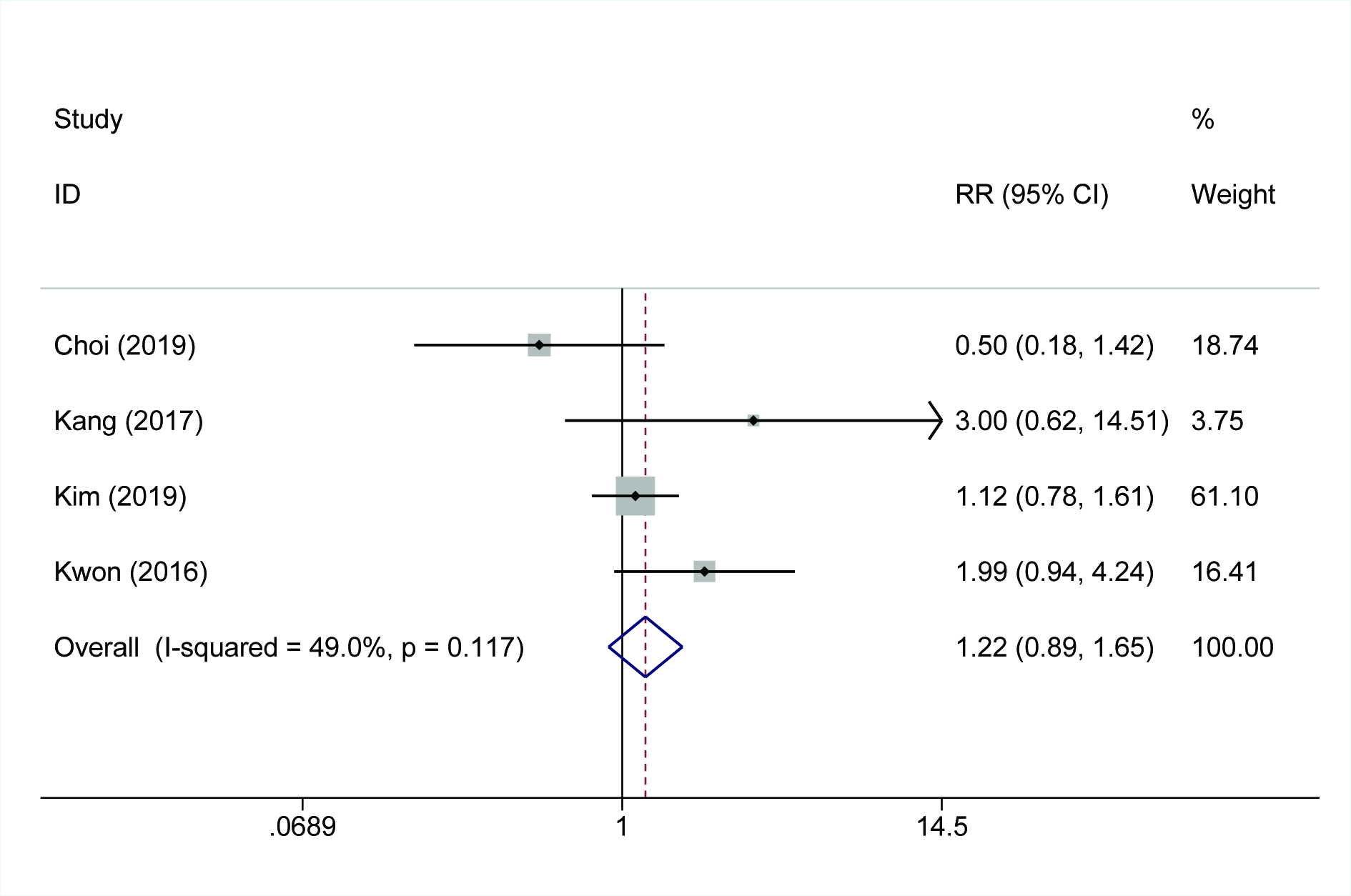
**Fig S5**


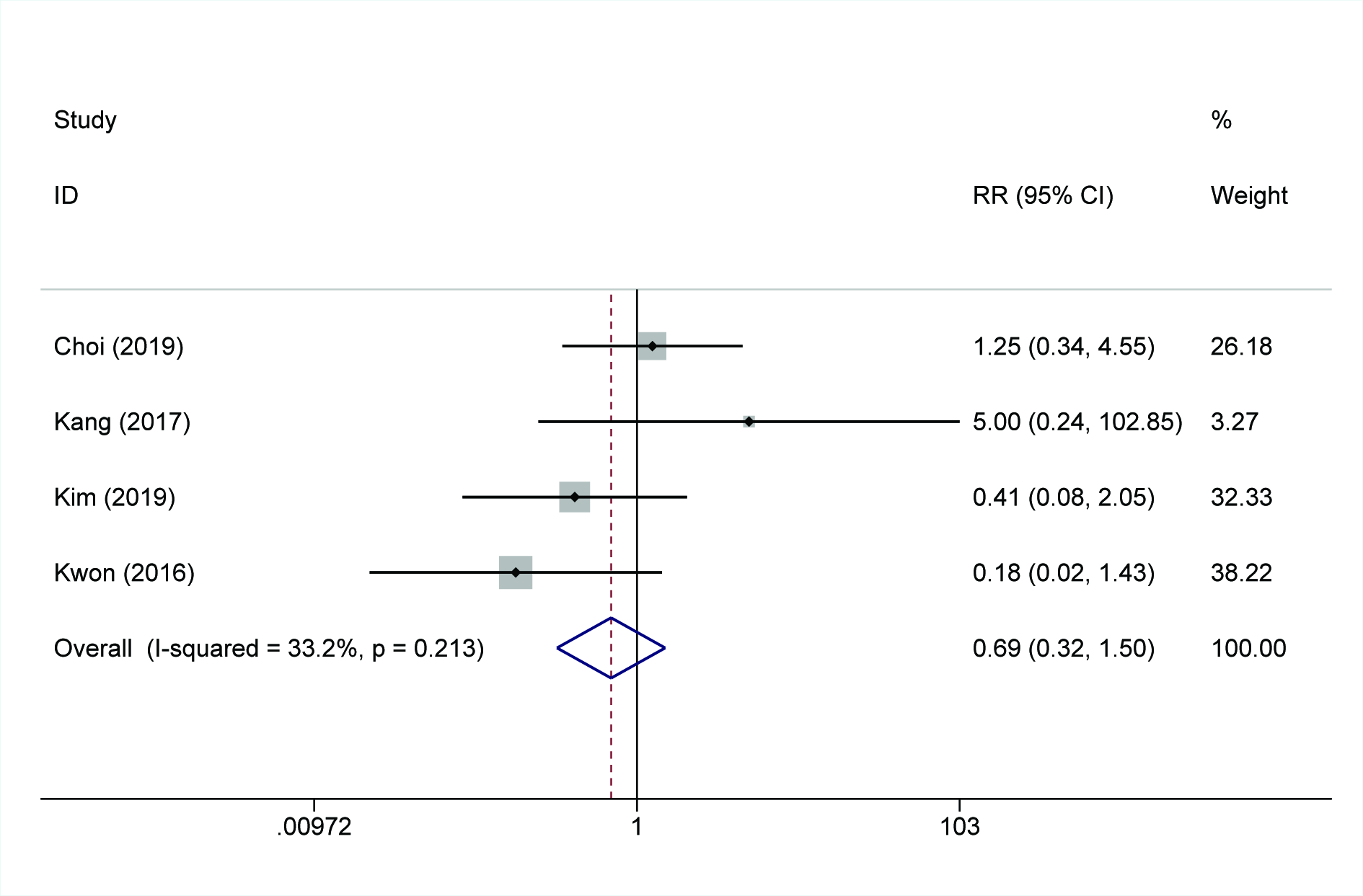
**Fig S6**


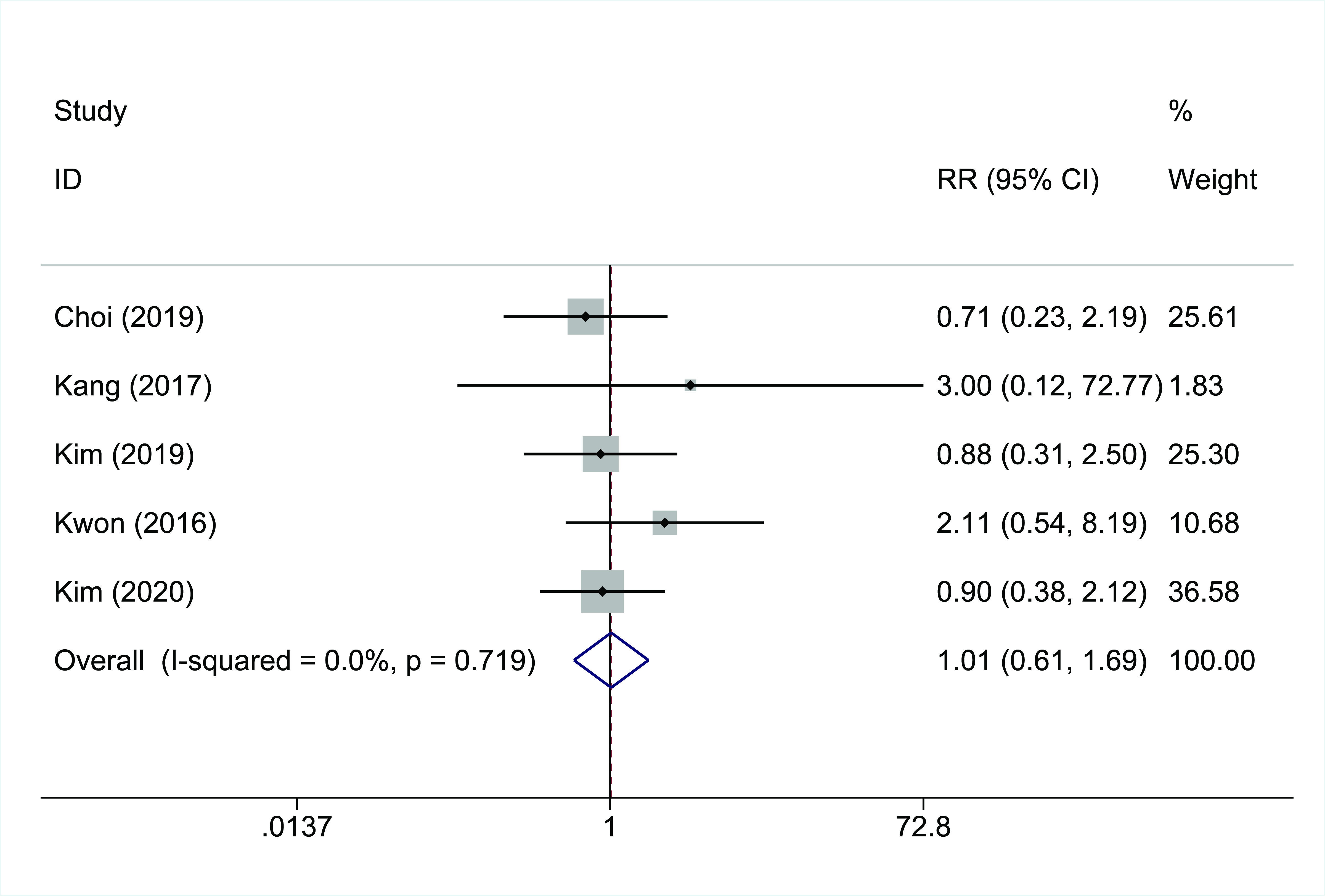
**Fig S7**


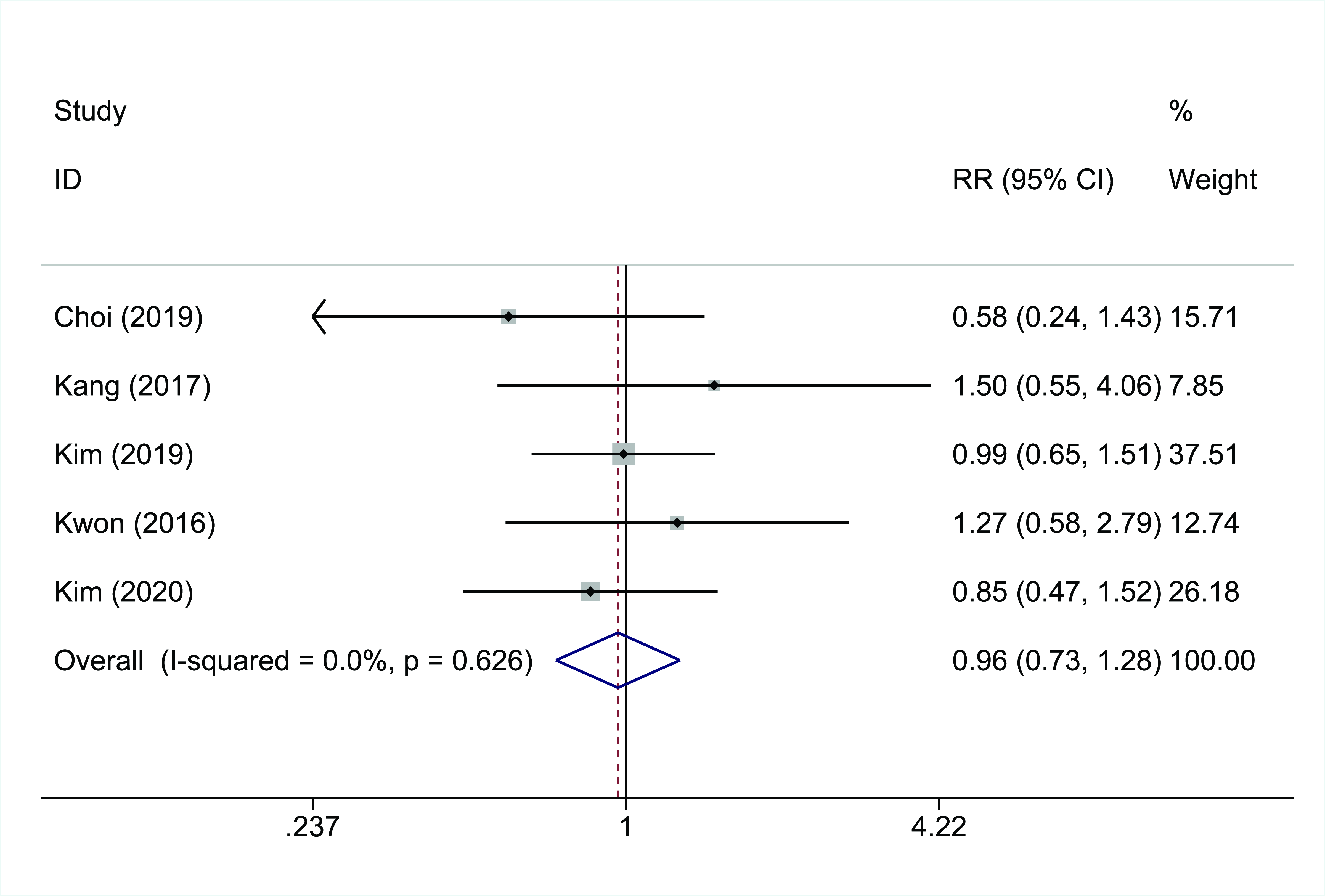
**Fig S8**
